# Comparative effect of bupivacaine local anesthesia with ropivacaine present with fentanyl

**DOI:** 10.6026/973206300210426

**Published:** 2025-03-31

**Authors:** Aman Gour, Vishwas Manohar Joshi, Jamale P.B

**Affiliations:** 1 Department of Anesthesiology, Krishna Institute of Medical Sciences, Krishna Vishwa Vidyapeeth (Deemed To Be University), Karad, Maharashtra, India

**Keywords:** Bupivacaine, ropivacaine, motor block, sensory block, fentanyl

## Abstract

Local anesthetics for lower limb anesthesia frequently use ropivacaine and bupivacaine due to their unique pharmacological properties. The point of this study was to look at how well efficacy of hyperbaric 0.5% bupivacaine with fentanyl and hyperbaric 0.75% ropivacaine with fentanyl worked in spinal anesthesia and compare and talk about them. Each group evaluated a total of 30 patients using various parameters to assess side effects and complications. We found among patients receiving bupivacaine, 70.0% did not require rescue medication, whereas 30.0% did. In contrast, among patients receiving ropivacaine, 86.7% did not require rescue medication, while 13.3% did. Thus, found non-significant difference. Bupivacaine has a faster onset and longer duration of sensory block and motor block, providing better early pain alleviation than ropivacaine.

## Background:

The International Association for the study of pain defines pain as an unpleasant sensory and emotional experience linked to actual or potential tissue damage. This phenomenon may result in central sensitization and facilitate the progression from acute to chronic pain [1]. A prominent and widely utilized method used all over the globe is spinal anesthesia, which is described as "the regional anesthesia obtained by blocking nerves in the subarachnoid space" [2]. The benefits of an awake patient ease of use, quick start of action, low medication cost, comparatively few side effects and rapid patient turnover have led many surgeons to choose this surgical technique. Both ropivacaine and bupivacaine are widely used local anesthetics with different pharmacological characteristics for lower limb anesthesia. Due to its lower cardiotoxicity than bupivacaine, ropivacaine is often chosen as a safer alternative for patients, particularly those who are at risk for cardiovascular problems [3]. Although ropivacaine tends to generate a less strong motor block, both anesthetics give excellent sensory and motor blockades. This may be helpful in postoperative recovery as it allows for earlier movement. Furthermore, the duration of action of ropivacaine is marginally less than that bupivacaine. Regarding analgesic efficacy, both medications demonstrate comparable effectiveness, delivering substantial pain relief. However, ropivacaine's lower limb solubility means that it is less likely to cause systemic toxicity. This makes it safer for longer procedures [4]. The incorporation of adjuvants like fentanyl into spinal anesthesia aims to decrease the necessary dosage of local anesthetics and facilitate a more rapid onset of sensory blockade. This combination additionally reduces the effective dosage of local anesthetics. Fentanyl acts as an agonist at the µ-opioid receptor, enhancing analgesic effects. Nonetheless, this may result in a higher frequency of adverse effects such as pruritus, urinary retention, nausea, vomiting and respiratory depression [5]. Therefore, it is of interest to report and compare 75% ropivacaine with fentanyl in sinoatrial patients undergoing lower limb surgeries.

## Materials and Methods:

We conducted a prospective, randomized clinical study over an 18-month period involving a total of 60 patients. Patients were randomly assigned to two groups, each consisting of 30 individuals. A pre-anesthetic evaluation was conducted, which included a physical examination, systemic assessment and laboratory investigations such as complete hemogram, random blood sugar, blood urea, serum creatinine, electrocardiogram, chest X-ray and coagulation profile. Patients were instructed to remain nil per os (nothing by mouth) for six hours prior to surgery and were administered 0.25 mg of oral alprazolam the night before the procedure, as well as on the day of surgery. (Group A) or experimental group, patients received sinoatrial with bupivacaine 0.5% 15 mg (3ml) with fentanyl 25 mcg(0.5ml) and Group B patients received sinoatrial with ropivacaine 0.75% 15mg (2ml) with fentanyl 25 mcg (0.5ml). Parameters included onset, time, degree and duration of sensory and motor block, hemodynamics changes (heart rate, blood pressure and respiratory rate at various intervals of intraoperative time) and side effects, complications (nausea, vomiting, shivering, hypotension, bradycardia and respiratory depression).

## Inclusion criteria:

[1] Age group between 18 to 60 years.

[2] American Society of Anesthesiologists grade I or II.

[3] Undergoing elective surgeries.

[4] Willing to participate.

## Exclusion criteria:

[1] Those who refused.

[2] Local infection like coagulpathies, spinal deformity, active disease of central nervous system, pre-existing motor or sensory deficit, allergy history to local anesthesia.

## Statistical analysis:

Unpaired student's test, Fisher's exact test, Mann-Whitney test, univariate analysis of variance and general linear model for repeated measures. P-values < 0.05 were considered statistically significant.

## Results:

Table 1 shows that, no statistically significant difference in the distribution of age groups between patients as indicated by a p-value of 0.281. Table 2 shows that, no statistically significant difference in the american society of anesthesiologist risk classification between patients as the p value of 0.706. Table 3 shows that, no statistically significant difference in the mean height of patients with a p-value of 0.580. Table 4 shows that, no statistically significant difference in the mean weight of patients using bupivacaine compared to those using ropivacaine, with a p-value of 0.089. Although the mean weight for the bupivacaine group (56.67 kg) is slightly higher than that for the ropivacaine group (52.97 kg), this difference is not significant at the conventional 0.05 threshold, indicating that it could be due to random variation rather than a real difference between the two groups. Table 5 shows that, bupivacaine and fentanyl (108.86 ± 11.945) and the other with ropivacaine and fentanyl (106.37 ±9.031). The overall mean duration for both groups combined is 107.59 ± 10.547. The minimum and maximum durations range from 90 to 132 minutes across the groups. The P value of 0.36 indicates that there is no statistically significant difference between the two groups. Table 6 shows that, mean onset time for Bupivacaine Fentanyl group is 3.433 ± 0.5040 and standard error 0.0920 minutes. For the Ropivacaine Fentanyl group, the mean onset time is significantly longer at 6.783± 0.9767 and standard error 0.1783 minutes. Thus P-value was less than 0.001, indicating statistically significant difference. Table 7 shows that, at correct Bupivacaine Fentanyl with Bupivacaine group, mean onset time was 4.77 ± 0.898 and standard error was 0.164 minutes. In contrast, the Ropivacaine Fentanyl group has a significantly longer mean onset time of 7.53±1.383 and standard error was 0.252 minutes. Thus P-value was less than 0.001, indicating a statistically significant. Table 8 shows that, at Bupivacaine Fentanyl group, mean duration was 333.000 ± 18.2757 minutes and standard error 3.3367 minutes. Correct Ropivacaine Fentanyl with ropivacaine group has a shorter mean duration of 292.433 ± 63.5793 minutes and standard error 11.6079 minutes as the P value was 0.001 indicates a statistically significant difference. Table 9 shows that, Bupivacaine Fentanyl group, mean duration was 301.900 ± 36.2314 minutes and SE was 6.6149 minutes. The Ropivacaine Fentanyl group, 263.800 ± 46.8530 minutes and standard error was 8.5541 minutes. Thus the P-value was 0.001 indicates a statistically significant difference. Table 10 shows that, significant differences in heart rate between the bupivacaine + fentanyl and ropivacaine + fentanyl groups at several time points. Specifically, the bupivacaine + fentanyl group has higher mean heart rate at 5 minutes (p = 0.0073), 15 minutes (p = 0.0307), 30 minutes (p = 0.0428), 60 minutes (p = 0.0050) and 120 minutes (p = 0.0199). No significant differences are observed at 0 minutes (p = 0.07), 10 minutes (p = 0.7132), 90 minutes (p = 0.8875) and 150 minutes (p = 0.8909). This suggests that bupivacaine tends to result in higher heart rates compared to ropivacaine during the early to mid-periods of the observation. Table 11 shows that, no statistically significant differences was seen as the p-values at 0 min was 0.184, 5 min was 0.627, 10 min was 0.075, 15 min was 0.738, 30 min was 0.907, 60 min was 0.271, 90 min was 0.903, 120 min was 0.211 and 150 min was 0.658 respectively. Table 12 shows that, no statistically significant differences as the p value at 0 min was 0.602, 5 min was 0.943, 10 min was 0.406, 30 min was 0.915, 60 min was 0.939, 90 min was 0.730, 120 min was 0.203 and 150 min was 0.182 respectively. Table 13 shows that, significant difference at 10 minutes, with bupivacaine resulting in higher systolic blood pressure compared to ropivacaine as the p value was 0.000. However, at all other time intervals at 0 min, 5 min, 30 min, 60 min, 90 min, 120 min and 150 min, there were no statistically significant differences observed in systolic blood pressure between the 2 groups (p > 0.05). This indicates that while bupivacaine may transiently elevate systolic blood pressure shortly after administration, this effect does not persist over the longer term and both anesthetics generally result in similar systolic blood pressure levels throughout the duration of measurement. Table 14 shows that, significant differences at 5 minutes as the p value was 0.004 and 10 minutes was 0.041 post-administration, with bupivacaine resulting in higher diastolic blood pressure compared to ropivacaine. However, no significant differences were found at 0 minutes, at 30 minutes, 60 minutes, 90 minutes, 120 minutes and 150 minutes post-administration (all p > 0.05). This indicates that while bupivacaine initially elevates diastolic blood pressure more than ropivacaine, these differences diminish over time, suggesting a similar effect on Diastolic blood pressure by both anesthetics in the longer term. Table 15 shows that, no significant differences in visual analog scores between the two groups at later time points (90 minutes, 120 minutes and 150 minutes), suggesting a convergence in pain relief efficacy over time. Table 16 shows that, both groups found no motor blockade (score of 0) at the initial and 5-minute marks. At 10 minutes, the bupivacaine group has a mean score of 0.10 ± 0.305 while the ropivacaine group remains at 0, with a significant P value with 0.042. At 15 minutes, the bupivacaine group has a mean score of 1.13 ± 0.346 and the Ropivacaine group a mean score of 1.00 ± 0.000, with a significant P value of 0.043. Both groups reach a full motor block (score of 2) by the 30-minute mark, maintained up to 150 minutes, with no significant differences as the P value was 0.00 respectively. Table 17 shows that, a higher proportion of patients in the bupivacaine group experienced adverse effects compared to those in the ropivacaine group. Specifically, the bupivacaine group had higher incidences of hypotension (20.0% vs. 0.0%), shivering (10.0% vs. 0.0%) and vomiting (13.3% vs. 0.0%) compared to the ropivacaine group. Table 18 shows that, no statistically significant difference in their distribution (χ^2^ = 3.706, p = 0.157). Among patients receiving bupivacaine, 70.0% did not require rescue medication, whereas 30.0% did. In contrast, among patients receiving ropivacaine, 86.7% did not require rescue medication, while 13.3% did.

## Discussion:

The data from our study suggests that the age distribution of the subjects in the Bupivacaine Fentanyl group is as follows: 6.7% are under the age of 20, 20.0% are between the ages of 21 and 25, 26.7% are between the ages of 25 and 30 and 46.7% are between the ages of 30 and 35. With 13.3% being under the age of 20, 30.0% being between the ages of 21 and 25, 33.3% being between the ages of 25 and 30 and 23.3% being between the ages of 30 and 35, this is the distribution of the Ropivacaine Fentanyl group. With a p-value of 0.281 and a Chi-Square value of 3.82, there is no statistically significant difference in the age distribution between the two groups. A meta-analysis of data from several randomized controlled trials and found that there was no significant difference in patient outcomes, including age distribution, when fentanyl was added to bupivacaine or ropivacaine [6]. Recent cohort studies identified analogous trends in age demographics among treatment groups, indicating that discrepancies, such as an increased proportion of patients aged 30-35 years in the bupivacaine group, are probably attributable to random sampling variability rather than a clinically significant difference. These results emphasize the need of rigorous statistical analysis, such as chi-square tests, in evaluating demographic data in clinical research to differentiate between genuine treatment effects and random chance fluctuations [7, 8]. A systematic review and other studies in this field have shown that bupivacaine and ropivacaine have similar safety profiles and American Society of Anesthesiologists risk distributions across a range of surgical procedure. The results showed that even though bupivacaine and ropivacaine have different pharmacokinetic properties and side effect profiles, they are both classified as having the same level of risk according to the American Society of Anesthesiologists (I, II and III) [9]. 2 other recent cohort studies, found that there were no significant differences in the distribution of American Society of Anesthesiologists when these local anesthetics were looked at [10, 11]. The differences in American Society of Anesthesiologists risk levels between treatment groups are likely due to random sampling error rather than big differences in the risk profiles of the patients, as shown by this data. Cohort studies have reported similar mean heights in patients receiving these treatments, suggesting that any differences noted are probably attributable to random sampling variability rather than representing a clinically significant difference [7-8]. Our study also found no statistically significant difference in mean weight between persons using bupivacaine and ropivacaine as the p value was 0.089. In our study, the analysis of mean surgical duration's patients receiving bupivacaine and fentanyl compared to those receiving ropivacaine and fentanyl indicates no statistically significant difference as the p value was 0.36. The average duration of surgery was compared between two groups: one receiving bupivacaine and fentanyl, with a mean duration of 108.86 minutes ± 11.945 and the other receiving ropivacaine and fentanyl with a mean duration of 106.37 minutes ± 9.031. The combined overall mean duration for both groups is 107.59 minutes ± 10.547 respectively. Research examining the onset times of sensory block has identified variations that are affected by the pharmacokinetic characteristics of these agents. It was shown in a systematic review that ropivacaine causes sensory block to start more quickly than bupivacaine during a variety of surgical procedures [9]. The results of the cohort studies showed that when ropivacaine is mixed with fentanyl, it usually blocks sensory pathways more quickly than bupivacaine [10, 11]. Another study concludes that the sequential administration of dexmedetomidine as an adjuvant with the local anaesthetic agent during the subarachnoid block enhances the onset of sensory and motor block, prolongs analgesia, diminishes overall analgesic requirements, increases patient satisfaction, and maintains stable hemodynamics compared to fentanyl. Bradycardia is common with dexmedetomidine [12]. A study shows that it works quickly and effectively to relieve pain, especially when mixed with other drugs like fentanyl, which makes it more useful in clinical settings. The results are very important for improving anesthetic protocols, especially for surgeries that need to start anesthesia quickly to make the patient more comfortable and speed up the process. The incorporation of these findings into clinical practice has the potential to enhance patient outcomes through the facilitation of more accurate and effective management of anesthesia onset times [13]. A study indicates that bupivacaine offers an extended duration of anesthesia attributable to its pharmacological characteristics, which are further augmented by the incorporation of fentanyl, an opioid known to extend analgesic effects [13]. A study also looks at the benefits of bupivacaine compared to ropivacaine. It focuses on the duration and strength of the sensory block, which makes it a better choice in many surgical situations [14]. A study showed that, due to its potent local anesthetic properties, bupivacaine generally provides a longer duration of both sensory and motor blocks compared to ropivacaine [15]. A study indicated that bupivacaine is linked to elevated heart rates during the initial phases of administration when compared to ropivacaine, probably as a result of its stronger sympathetic nervous system blockade. Therefore, this phenomenon may result in a compensatory elevation in heart rate to sustain cardiac output [16]. A study showed that both anesthetics keep mean arterial blood pressure levels about the same during surgery. This is because they are both amide-type local anesthetics with similar pharmacodynamics properties. Although there are variations in potency and onset times, their impact on blood pressure tends to be comparable in clinical settings when dosages are correctly administered [17]. The findings of the present study are further supported by a review, which highlights the fact that the addition of fentanyl to local anesthetics like bupivacaine and ropivacaine does not significantly change Mean arterial pressure [18]. In our study, on comparing systolic blood pressure between patients receiving bupivacaine with fentanyl and ropivacaine with fentanyl demonstrates a significant difference at the 10-minute mark, with the visual analog scores group exhibiting higher systolic blood pressure as the p value was 0.000. However, at all other time intervals (0 min, 5 min, 30 min, 60 min, 90 min, 120 min and 150 min), there were no statistically significant differences in systolic blood pressure between the two groups (p > 0.05). This indicates that bupivacaine may transiently elevate systolic blood pressure shortly after administration, but this effect does not persist over time, resulting in similar systolic blood pressure levels between the two anesthetics during the longer measurement periods. Research indicates that the administration of different local anesthetics can produce a range of effects on blood pressure parameters. However, because bupivacaine has a stronger affinity for cardiac sodium channels, it can cause early sympathetic activation, which can cause Blood Pressure to rise soon after administration [9]. Studies indicate that both anesthetics completely block motor functions within 30 minutes about the same amount of time [18, 19]. Another research found that, bupivacaine is more likely to cause side effects like low blood pressure, shivering and nausea or vomiting than ropivacaine. This is because bupivacaine is more likely to dissolve in lipids and cause systemic toxicity. The adverse effects associated with Bupivacaine are primarily linked to its increased likelihood of causing cardiovascular depression and central nervous system effects, particularly when administered at elevated doses or in susceptible patient groups [6]. Researches indicate that, bupivacaine offers a strong and prolonged analgesic effect. However, its administration may result in an increased need for rescue analgesia in certain situations, particularly when the initial block does not adequately cover extended procedures. Conversely, ropivacaine demonstrates a comparatively safer cardiovascular profile, leading to a reduced requirement for rescue medication, which indicates its stability and efficacy over an extended period [17, 19]. The absence of a statistically significant difference in the present study suggests that both anesthetic agents are broadly effective in pain management, while individual patient responses may differ according to particular clinical situations and procedural requirements. This information assists anesthesiologists in selecting between Bupivacaine and Ropivacaine, taking into account the desired balance of potency, side effect profile and the necessity for supplementary analgesic interventions during and post-surgery. A study has showed that, opioids as adjuvants to intrathecal bupivacaine are a commonly used intervention to achieve good postoperative outcomes. We conclude that both fentanyl and nalbuphine were equally efficacious in providing excellent intraoperative surgical anaesthesia and postoperative analgesia with good hemodynamic stability. The fentanyl group had a faster onset of both sensory and motor blockade as compared to the nalbuphine group, though it was not statistically significant. The duration of effective analgesia was, however, significantly more in the nalbuphine group [20]. A study done by Canan et al. concluded through their study that the elective cesarean delivery, the combinations of bupivacaine + fentanyl or ropivacaine + fentanyl exhibited similar anesthetic efficacy and fetal and maternal effects [21].

## Conclusion:

Bupivacaine demonstrates a quicker onset and extended duration of both sensory and motor blocks, offering superior early pain relief in comparison to ropivacaine. Nonetheless, it is associated with increased occurrences of adverse effects, including hypotension, shivering and vomiting. In spite of the differences that have been seen, both anesthetics have similar effects on oxygen saturation level, Mean arterial pressure and overall hemodynamic stability. There are also no significant differences in the need for rescue medication.

## Aim:

To compared and evaluated the efficacy of hyperbaric 0.5% bupivacaine with fentanyl and hyperbaric 0.75% ropivacaine with fentanyl in sinoatrial, in patients undergoing lower limb surgeries.

## Figures and Tables

**Table 1:** Age distribution

**Age group**		**Group**					**Chi Square (p value)**
	**Bupivacaine**			**Ropivacaine**			
	**Count**		**Column N %**	**Count**		**Column N %**	
<20 years		2	6.70%		4	13.30%	3.82 (0.281)
21-25 years		6	20.00%		9	30.00%	
25-30 years		8	26.70%		10	33.30%	
30-35 years		14	46.70%		7	23.30%	
total		30	100		30	100	

**Table 2:** American society of anesthesiologist's status

**American Society of Anesthesiologists Risk**			**Group**				**Chi Square (p value)**
	**Bupivacaine**			**Ropivacaine**			
	**Count**		**Column N %**	**Count**		**Column N %**	
I		9	30.00%		12	40.00%	0.696 (0.706)
II		17	56.70%		15	50.00%	
III		4	13.30%		3	10.00%	

**Table 3:** Height distribution

	**Group**	**N**	**Mean**	**Std. Deviation**	**t test**	**p value**
Height	Bupivacaine	30	159.57	5.01	0.556	0.58
	Ropivacaine	30	158.87	4.74	0.556	0.58

**Table 4:** Weight distribution

	**Group**	**N**	**Mean**	**Std. Deviation**	**t test**	**p value**
Weight	Bupivacaine	30	56.67	8.91	1.732	0.089
	Ropivacaine	30	52.97	7.59	1.732	0.089

**Table 5:** Mean duration of surgery (mm)

Group	Mean		Std. Deviation	Minimum	Maximum	P value
Bupivacaine	108.86	29	11.945	91	132	0.36
Ropivacaine	106.37	30	9.031	90	120	
Total	107.59	59	10.547	90	132	

**Table 6:** Time of onset of Sensory

	**GRP**	**N**	**Mean**	**Std. Deviation**	**t test**	**p value**
Time of onset of Sensory(TOS) Block at T10(min)	Bupivacaine			30	3.433	0.504	0.092	<0.001
	Ropivacaine			30	6.783	0.9767	0.1783	

**Table 7:** Time of onset of motor

	**GRP**	**N**	**Mean**	**Std. Deviation**	**t test**	**p value**
Time of onset of motor Block(TOM) at T10(min)	Bupivacaine		30	4.77	0.898	0.164	<0.001
	Ropivacaine		30	7.53	1.383	0.252	

**Table 8:** Mean duration (MD) SB (min)

	**GRP**	**N**	**Mean**	**Std. Deviation**	**Std. Error Mean**	**p value**
Duration of sensory block at T10 (min)	Bupivacaine		30	333	18.2757	3.3367	0.001
	Ropivacaine		30	292.433	63.5793	11.6079	

**Table 9:** Duration of motor block

	**GRP**	**N**	**Mean**	**Std. Deviation**	**Std. Error Mean**	**p value**
Duration of motor block(DMB) at T 10(min)	Bupivacaine		30	301.9	36.2314	6.6149	0.001
	Ropivacaine		30	263.8	46.853	8.5541	

**Table 10:** Heart rate distribution

**Heart Rate**	**Group**	**N**	**Mean**	**Std. Deviation**	**t test**	**p value**
0 min	Bupivacaine	30	85.2	9.45	1.846	0.07
	Ropivacaine	30	87.56	9.82		
5 min	Bupivacaine	30	82.4	13.44	2.7792	0.0073
	Ropivacaine	30	74.17	9.1		
10 min	Bupivacaine	30	84.67	7	0.3693	0.7132
	Ropivacaine	30	84	6.98		
15 min	Bupivacaine	30	84.57	8.42	2.2148	0.0307
	Ropivacaine	30	79.63	8.83		
30 min	Bupivacaine	30	92.2	9.37	2.0712	0.0428
	Ropivacaine	30	87.13	9.58		
60 min	Bupivacaine	30	82.53	12.56	2.9162	0.005
	Ropivacaine	30	74.27	9.13		
90 min	Bupivacaine	30	84.4	6.83	0.1421	0.8875
	Ropivacaine	30	84.13	7.68		
120 min	Bupivacaine	30	83.83	6.52	2.3945	0.0199
	Ropivacaine	30	79.3	8.06		
150 min	Bupivacaine	30	80.63	12.79	-0.1378	0.8909
	Ropivacaine	30	81.07	11.55		

**Table 11:** Oxygen saturation level

**SPO2**	**Group**	**N**	**Mean**	**Std. Deviation**	**t test**	**p value**
0 min	Bupivacaine	30	98.53	1.14	1.345	0.184
	Ropivacaine	30	98.13	1.17		
5 min	Bupivacaine	30	98.43	1.07	-0.489	0.627
	Ropivacaine	30	98.57	1.04		
10 min	Bupivacaine	30	98.93	1.08	1.816	0.075
	Ropivacaine	30	98.4	1.19		
15 min	Bupivacaine	30	98.47	1.07	-0.336	0.738
	Ropivacaine	30	98.57	1.22		
30 min	Bupivacaine	30	98.3	1.12	0.117	0.907
	Ropivacaine	30	98.27	1.08		
60 min	Bupivacaine	30	98.53	1.14	1.111	0.271
	Ropivacaine	30	98.2	1.19		
90 min	Bupivacaine	30	98.47	1.11	0.122	0.903
	Ropivacaine	30	98.43	1.01		
120 min	Bupivacaine	30	98.87	1.07	1.266	0.211
	Ropivacaine	30	98.5	1.17		
150 min	Bupivacaine	30	98.33	1.09	-0.445	0.658
	Ropivacaine	30	98.47	1.22		

**Table 12:** Mean arterial pressure distribution

**MAP**	**Group**	**N**	**Mean**	**Std. Deviation**	**t test**	**p value**
0 min	Bupivacaine	30	89.83	3.83	-0.525	0.602
	Ropivacaine	30	90.37	4.04		
5 min	Bupivacaine	30	77.7	14.47	-0.071	0.943
	Ropivacaine	30	77.97	14.46		
10 min	Bupivacaine	30	90.1	4.41	0.836	0.406
	Ropivacaine	30	88.87	6.77		
5 min	Bupivacaine	29	89.97	5.23	0.287	0.775
	Ropivacaine	30	89.53	6.27		
30 min	Bupivacaine	30	80.57	13.35	-0.108	0.915
	Ropivacaine	30	80.9	10.47		
60 min	Bupivacaine	30	90.37	4.93	0.077	0.939
	Ropivacaine	30	90.27	5.13		
90 min	Bupivacaine	30	90.43	4.45	0.347	0.73
	Ropivacaine	30	89.83	8.37		
120 min	Bupivacaine	30	75.63	14.47	-1.288	0.203
	Ropivacaine	30	80.33	13.79		
150 min	Bupivacaine	30	78.6	13.71	-1.352	0.182
	Ropivacaine	30	83.2	12.63		

**Table 13:** Systolic blood pressure distribution

**Systolic Blood Pressure**	**Group**	**N**	**Mean**	**Std. Deviation**	**t test**	**p value**
0 min	Bupivacaine	30	119.23	8	-0.288	0.774
	Ropivacaine	30	119.83	8.13		
5 min	Bupivacaine	30	87.17	8.03	1.003	0.32
	Ropivacaine	30	85.4	5.33		
10 min	Bupivacaine	30	105.83	17.15	5.335	0
	Ropivacaine	30	88.33	5.36		
5 min	Bupivacaine	30	140.17	183.93	0.849	0.4
	Ropivacaine	30	111.6	13.06		
30 min	Bupivacaine	30	108.43	16.39	-1.031	0.307
	Ropivacaine	30	112.43	13.53		
60 min	Bupivacaine	30	115.1	15.81	0.622	0.536
	Ropivacaine	30	112.9	11.17		
90 min	Bupivacaine	30	113.3	12.92	-0.384	0.703
	Ropivacaine	30	114.4	8.94		
120 min	Bupivacaine	30	111.2	8.6	-1.202	0.234
	Ropivacaine	30	114.4	11.77		
150 min	Bupivacaine	30	112.67	10.37	-0.91	0.367
	Ropivacaine	30	115.13	10.64		

**Table 14:** Diastolic blood pressure distribution

**Diastolic Blood Pressure**	**Group**	**N**	**Mean**	**Std. Deviation**	**t test**	**p value**
0 min	Bupivacaine	30	74.6	5.26	-0.216	0.829
	Ropivacaine	29	74.97	7.55		
5 min	Bupivacaine	30	60.03	7.18	2.957	0.004
	Ropivacaine	30	55.03	5.85		
10 min	Bupivacaine	30	64.07	12.22	2.087	0.041
	Ropivacaine	30	58.87	6.08		
5 min	Bupivacaine	30	63.47	11.26	-1.892	0.064
	Ropivacaine	30	69.07	11.66		
30 min	Bupivacaine	30	64.27	14.06	-1.506	0.137
	Ropivacaine	30	69.5	12.82		
60 min	Bupivacaine	30	64.67	12.74	-0.669	0.506
	Ropivacaine	30	66.9	13.12		
90 min	Bupivacaine	30	65.4	10.12	-0.579	0.565
	Ropivacaine	30	67.13	12.91		
120 min	Bupivacaine	30	66	9.13	-1.393	0.169
	Ropivacaine	30	69.6	10.82		
150 min	Bupivacaine	30	65.97	9.28	-1.43	0.158
	Ropivacaine	30	69.73	11.05		

**Table 15:** Visual analog scores distribution

**visual analog scores**	**Group**	**N**	**Mean**	**Std. Deviation**	**t test**	**p value**
0 min	Bupivacaine	30	7.8	1.1	0.377	0.707
	Ropivacaine	30	7.7	0.95		
5 min	Bupivacaine	30	1.6	0.67	-2.53	0.014
	Ropivacaine	30	2.13	0.94		
10 min	Bupivacaine	30	1.5	0.51	-3.084	0.003
	Ropivacaine	30	2.07	0.87		
15 min	Bupivacaine	30	1.5	0.51	-3.153	0.003
	Ropivacaine	29	2.07	0.84		
30 min	Bupivacaine	30	1.53	0.51	-4.062	0
	Ropivacaine	29	2.45	1.12		
60 min	Bupivacaine	30	1.67	0.55	-10.741	0
	Ropivacaine	29	5.76	2.01		
90 min	Bupivacaine	30	2.3	0.92	-0.976	0.333
	Ropivacaine	30	2.53	0.94		
120 min	Bupivacaine	30	5.03	1.71	1.392	0.169
	Ropivacaine	30	4.4	1.81		
150 min	Bupivacaine	29	6.31	1.17	-0.194	0.847
	Ropivacaine	30	6.37	1.07		

**Table 16:** Modified bromage scale

	**GROUP**	**N**	**Mean**	**Std. Deviation**	**p value**
MODIFIED BROMAGE	Bupivacaine	30	0	.000a	0.078
SCALE at 0	Ropivacaine	30	0	.000a	
MODIFIED BROMAGE	Bupivacaine	30	0	.000a	0.083
SCALE 5 MIN AFTR BLK	Ropivacaine	30	0	.000a	
MODIFIED BROMAGE	Bupivacaine	30	0.1	0.305	0.042
SCALE 10 MIN	Ropivacaine	30	0	0	
MODIFIED BROMAGE	Bupivacaine	30	1.13	0.346	0.043
SCALE 15 MIN	Ropivacaine	29	1	0	
MODIFIED BROMAGE	Bupivacaine	30	2	.000a	0
SCALE 30 MIN	Ropivacaine	30	2	.000a	
MODIFIED BROMAGE	Bupivacaine	30	2	.000a	0
SCALE 60 MIN	Ropivacaine	30	2	.000a	
MODIFIED BROMAGE	Bupivacaine	30	2	.000a	0
SCALE 120 MIN	Ropivacaine	30	2	.000a	
MODIFIED BROMAGE	Bupivacaine	30	2	.000a	0
SCALE 150 MIN	Ropivacaine	30	2	.000a	

**Table 17:** Adverse effects

**ADVERSE EFFECTS**			**GROUP**				**CHI SQUARE (P VALUE)**
	**Bupivacaine**			**Ropivacaine**			
	**Count**		**Column N %**	**Count**		**Column N %**	
None		13	43.30%		25	83.30%	22.78 (0.002)
Bradycardia		3	10.00%		4	13.30%	
Bradycardia, Hypotension		1	3.30%		0	0.00%	
Drowsiness		0	0.00%		1	3.30%	
Hypotension		6	20.00%		0	0.00%	
Shivering		3	10.00%		0	0.00%	
Vomiting		4	13.30%		0	0.00%	

**Table 18:** Rescue analgesia

**Rescue medication**			**GROUP**				**CHI SQUARE (P VALUE)**
	**Bupivacaine**			**Ropivacaine**			
	**Count**		**Column N %**	**Count**		**Column N %**	
No		21	70.00%		26	86.70%	3.706 (0.157)
Yes		9	30.00%		4	13.30%	

## References

[1] Majedi H (2019). Anesthesiology and pain medicine..

[2] Vadivelu N (2014). Local and regional anesthesia..

[3] Hernandez A.N, Hendrix J.M (2024). Epidural Anesthesia..

[4] Bhat S.N, Upadya M (2013). Anesthesia Essays and Researches..

[5] Swain A (2017). World journal of clinical cases..

[6] Smith G (2025). General Anesthesia for Surgeons.

[7] Johnson R.L (2017). J Bone Joint Surg Am..

[8] Lee S (2024). Anesthesia & Analgesia..

[9] Brown C.A (2023). Annals of Plastic Surgery..

[10] Garcia D (2022). Cureus..

[11] Patel B.J (2023). Cureus..

[12] Shukla U (2024). Cureus..

[13] Kuthiala G, Chaudhary G (2011). Indian journal of anaesthesia..

[14] Bajwa S.J, Kaur J (2013). Journal of Anaesthesiology Clinical Pharmacology..

[15] Whiteside J.B (2003). British journal of anaesthesia..

[16] Graf B.M (2002). The Journal of the American Society of Anesthesiologists..

[17] McClellan K.J, Faulds D (2000). Drugs..

[18] Vercauteren M.P (2001). Anesthesia & Analgesia..

[19] Casati A, Baciarello M (2006). Current Drug Therapy..

[20] Satapathy S (2023). Cureus..

[21] Canan U (2013). Nigerian journal of clinical practice..

